# Special Issue “Breast Cancer: From Pathophysiology to Novel Therapies”

**DOI:** 10.3390/ijms27125238

**Published:** 2026-06-10

**Authors:** Jennifer Sims-Mourtada

**Affiliations:** Cawley Center for Translational Cancer Research, Helen F Graham Cancer Center and Research Institute, Christiana Care Health Services, Inc., 4701 Ogletown Stanton Rd Suite 4300, Newark, DE 19713, USA; jsimsmourtada@christianacare.org; Tel.: +1-302-623-4648; Fax: +1-302-623-4314

Breast cancer is among the most common malignancies worldwide and remains a leading cause of cancer-related mortality in women [1]. Despite substantial advances in early detection and treatment, outcomes remain highly variable, reflecting the profound biological diversity of the disease. While many patients with hormone receptor-positive (HR+) breast cancer experience favorable outcomes with endocrine-based therapies, a significant subset develops resistance, leading to early relapse or late recurrences that may occur from years to decades after the initial diagnosis [2]. Triple-negative breast cancer (TNBC), defined by the absence of estrogen receptors (ERs), progesterone receptors (PRs), and HER2 expression, is associated with aggressive clinical behavior, early recurrence, and limited therapeutic options. Although recent advances, including immune checkpoint inhibitors and antibody–drug conjugates, have improved outcomes in TNBC, these gains remain incremental, with benefits in progression-free survival typically measured in months [3,4]. While these advances represent meaningful improvements over standard chemotherapy, they do not fundamentally alter the natural history of metastatic breast cancer for many patients, as resistance inevitably develops.

## 1. Limitations of Receptor-Based Classification

Although breast cancer has traditionally been identified and treated based on receptor status, these classifications do not represent biologically uniform diseases, nor do they reliably predict long-term outcomes. Within each receptor-defined subtype, substantial heterogeneity influences treatment response, risk of recurrence, and survival [5]. Patients with seemingly similar tumors based on histopathology can experience markedly different clinical trajectories, underscoring the limitations of this framework.

At the molecular level, breast cancer encompasses multiple transcriptional and biological subtypes [6,7]. Molecular classifications such as basal-like, mesenchymal, immunomodulatory, and luminal androgen receptor-associated phenotypes, each defined by distinct signaling pathways, immune landscapes, and metabolic dependencies [7]. Even within these molecular classes, substantial heterogeneity likely exists, contributing to the variable clinical responses observed and representing a central barrier to achieving durable disease control.

This Special Issue of IJMS, Breast Cancer: From Pathophysiology to Novel Therapies, reports the continued discovery of breast cancer heterogeneity and highlights the need for a deeper and more integrated understanding of breast cancer biology to advance the development of more effective therapeutic strategies. Collectively, these six original research articles and four review articles emphasize that breast cancer is a highly adaptive and heterogeneous disease shaped by complex interactions between the regulation of gene expression, metabolism, signaling pathways, and the tumor microenvironment. The issue further underscores the importance of developing advanced experimental models that more accurately recapitulate human tumor complexity, including patient-derived organoids, multi-omics platforms, and microenvironment-focused systems.

## 2. MicroRNA-Driven Regulation of Signaling Heterogeneity

Heterogeneity in gene expression is a defining feature of breast cancer and contributes substantially to differences in tumor behavior, therapeutic response and clinical outcome [8]. MicroRNAs are dynamic regulators of gene expression in breast cancer [9,10]. Unlike genetic alterations, microRNAs can simultaneously regulate multiple downstream targets and pathways, allowing tumors to adapt to environmental stressors and therapeutic pressures [11]. Regulation by microRNAs at the genetic and signaling level is not fixed, but may be context-dependent, contributing to tumor heterogeneity [12]. In this special addition, Krol-Jatrega et al. demonstrate coordinated downregulation of tumor suppressors (TP53, MAP3K1, MAP2K4) alongside upregulation of oncogenic mediators (TGFB1, PPM1D), highlighting network-level dysregulation rather than isolated mutations (Contribution 1). Importantly, this study showed that microRNAs directly regulate these pathways by modulating gene expression, enabling tumors to adapt signaling networks. Complementary analyses of the TGFβ/SMAD axis (Contribution 2) further demonstrate that microRNA-associated dysregulation contributes to overexpression of SMAD3, SMAD4, and SMAD5, alongside reduced SMAD7, promoting pro-tumorigenic signaling and reinforcing pathway-level adaptation. Emerging evidence also suggests that microRNA regulation contributes to population-level disparities in breast cancer (Contribution 3). Distinct microRNA expression patterns have been shown to differentially regulate androgen receptor signaling in AR-negative or quadruple-negative TNBC, a subtype more prevalent among women of African descent and associated with poorer outcomes. These findings indicate that microRNAs not only modulate core signaling pathways but also shape subtype biology and disease aggressiveness across populations. Together, these studies position microRNAs as central regulators of signaling heterogeneity, enabling tumors to engage distinct regulatory circuits while converging on similar functional outcomes.

## 3. Metabolic Heterogeneity and Tumor Adaptation

Beyond signaling networks, metabolic reprogramming is increasingly recognized as a critical driver of tumor progression and resistance [13,14]. Breast cancers exhibit substantial metabolic plasticity enabling adaptation to hypoxia, oxidative stress, nutrient deprivation and therapeutic pressure [15,16]. Importantly, these metabolic states are not uniform across breast cancer or even within individual tumors, contributing to tumor heterogeneity [13,17]. In TNBC, these metabolic alterations are particularly pronounced, with tumors exhibiting enhanced glycolysis, altered mitochondrial respiration and increased dependence on redox balance to sustain aggressive growth and metastatic behavior [18]. Bocachica-Adorno et al., (Contribution 4), report that ergosterol peroxide (EP), a natural compound derived from Ganoderma Lucidum, exploits oxidative stress vulnerabilities in TNBC by disrupting mitochondrial membrane potential, reducing oxidative phosphorylation. This study further demonstrates that EP significantly decreases tumor growth and metastasis, highlighting the therapeutic potential of targeting metabolic and mitochondrial dependencies in aggressive TNBC. These findings support the broader concept that manipulation of redox balance and metabolic stress may sensitize tumors to treatment. However, in contribution Ajayi et al. (Contribution 5) demonstrates that these dependencies may not be uniform across all tumor cells. This study shows that deletion of the stem cell marker ALDHA1A1, a key regulator of redox homeostasis and stemness, increases oxidative stress and alters therapeutic response in only select clonal populations, emphasizing that metabolic and redox vulnerabilities can vary substantially even within the same tumor.

Tumor metabolism may not only support tumor growth but may also have an effect on the immune microenvironment. In Contribution 6, Avalos-Navarro et al. review how enhanced fatty acid oxidation and glycogen metabolism support tumor survival under stress and are tightly linked to immune suppression, in part through the promotion of macrophage polarization and the inhibition of effective anti-tumor immune responses. Collectively, these findings underscore that metabolic heterogeneity is not merely a feature of tumor adaptation but a driver of microenvironmental remodeling, reinforcing that effective therapeutic strategies must account for the immunomodulatory effects of tumor metabolism rather than targeting isolated metabolic pathways.

## 4. Tumor Microenvironment and Immune Interactions

Tumor progression and therapeutic response are not determined solely by the intrinsic properties of tumor cells but are profoundly influenced by interactions within the tumor microenvironment. Increasing evidence demonstrates that immune cells, stromal components, systemic metabolism, and inflammatory signaling collectively shape tumor evolution, therapeutic resistance, and metastatic progression [19,20]. In this Special Issue, three review articles highlight the complex and dynamic interactions between breast tumors and their surrounding microenvironment, emphasizing how immune regulation, metabolic adaptation, and host-specific factors contribute to breast cancer heterogeneity and treatment response. Avalos-Navarro et al. highlight that immune heterogeneity in breast cancer is closely intertwined with metabolic adaptation, where tumor-driven shifts in glycolysis, fatty acid oxidation, glutamine metabolism, and glycogen utilization actively shape the immune microenvironment. Their review describes how these metabolic programs promote immunosuppressive macrophage polarization, neutrophil recruitment and NET formation, T cell dysfunction, and inflammatory signaling pathways that collectively support metastatic progression and therapeutic resistance. These findings reinforce the growing recognition that tumor metabolism and immune regulation are highly interconnected processes that contribute to the diverse biological behavior observed across breast cancers. While the immune microenvironment is most often investigated in TNBC, which are often rich in tumor-infiltrating lymphocytes [21], two papers in this Special Issue highlight the role of the immune microenvironment in HR+ tumors (Contributions 7 and 8). Despite being considered immunologically “cold,” HR+ tumors can exhibit context-dependent immune activation, and emerging strategies aimed at reprogramming the tumor microenvironment have demonstrated that even these tumors are more dynamic than previously appreciated. Lin et al. review the literature showing that HR+ breast cancers are not uniformly immunologically inert but instead contain biologically heterogeneous and potentially immune-responsive subsets. The authors detail how chemotherapy-induced immunogenic cell death, checkpoint blockade and tumor microenvironment remodeling collectively contribute to immune activation in HR+ breast cancer. In addition, Tang et al. comprehensively review the evolving role of immune checkpoint inhibition in HR+ breast cancer. This review emphasizes strategies to convert immunologically cold tumors into more responsive hot tumors through different treatment combinations, including chemotherapy, CDK4/6 inhibitors, PARP inhibitors, and external beam radiation treatment. This review further highlights biomarkers of immune response in HR+ cancers, such as tumor mutational burden, immune subsets and gut microbiome signatures. The role of systemic and patient-specific factors in shaping immune responses and therapeutic efficacy is further explored in Contribution 9. Cheng et al. demonstrate that variations in body composition, including adiposity and skeletal muscle mass, are associated with distinct patterns of immune cell infiltration and PI3K/AKT signaling, suggesting that host physiology can directly influence tumor biology and therapeutic response. These findings underscore that resistance is not solely a property of tumor cells but emerges from the coordinated interaction between tumor, immune system, and host environment.

## 5. Advancing Experimental Models to Capture Breast Cancer Heterogeneity

A recurring theme across these studies is the inadequacy of current experimental models to capture the full spectrum of tumor heterogeneity. Traditional systems, including cell lines and xenografts, fail to replicate the complexity of human tumors, limiting their predictive value. Advances in patient-derived organoids, organ-on-chip technologies, and multi-omics approaches, which provide more representative platforms that better preserve tumor architecture, cellular diversity, and microenvironmental interactions [22,23], are reviewed in Contribution 10. These models are not merely technical improvements but are essential for identifying clinically relevant vulnerabilities and for designing combination therapies that reflect the multifactorial nature of tumor biology.

## 6. Conclusions

The studies in this issue make a compelling case that the persistent gap between therapeutic innovation and durable clinical benefit in breast cancer is not due to a lack of targets, but rather due to tumor complexity. Breast cancer does not fail treatment because it lacks vulnerabilities; it fails because those vulnerabilities are heterogeneous, context-dependent, and dynamically regulated. Although receptor-based subtyping has transformed the clinical management of breast cancer, these categories often oversimplify the underlying biology and fail to account for the extensive transcriptional, metabolic, immune, and microenvironmental diversity that exists both between patients and within individual tumors. As such, approaches that target single pathways or rely on static subtype definitions are unlikely to succeed. Instead, progress will depend on embracing breast cancer as a multi-dimensional, adaptive system, in which genetic, metabolic, and microenvironmental factors must be addressed simultaneously.
